# Students’ perceptions of general medicine following community‐based medical education in rural Japan

**DOI:** 10.1002/jgf2.274

**Published:** 2019-09-08

**Authors:** Ryuichi Ohta, Yoshinori Ryu, Takuji Katsube, Yoshihiro Moriwaki, Jun Otani

**Affiliations:** ^1^ Community Care Unnan City Hospital Unnan Japan; ^2^ Surgery Unnan City Hospital Unnan Japan

**Keywords:** community‐based medical education, community‐based medicine, family practice, medical education, primary care, rural medicine

## Abstract

**Background:**

Japan's population is rapidly aging, and at the same time, the number of medical students interested in general or family medicine is declining. Community‐based medical education (CBME) programs may be used to promote interest and competencies in general medicine among medical students.

**Method:**

This mixed‐method study investigated the perceptions of fifth‐ and sixth year undergraduate medical students who completed a two week CBME course in Unnan, a small city in rural Japan. The participants completed two survey questionnaires: (a) The achievement questionnaire administered pre‐ and posttraining, and (b) the curriculum content questionnaire administered posttraining. To understand the students’ perceptions about general medicine further, semistructured interviews were conducted with each participant post‐CBME training.

**Results:**

The participants’ ratings on the achievement survey improved significantly from pre‐ to posttraining. The average ratings for the curriculum content survey indicated that the educational objectives were met in all but one area. A qualitative analysis of the interview data revealed that the participants had little exposure to general medicine at their university hospital, and there was a lack of understanding in other medical professionals regarding the roles of general medicine physicians.

**Conclusion:**

This study demonstrates that there are educational gaps between medical universities and community hospitals regarding general medicine. Increased exposure, early exposure, and a clarification of the competencies were noted as areas to improve the students’ understanding of general medicine. Undergraduates should be exposed to general medicine more frequently and from early training stages through effective collaborations between universities and hospitals.

## 1 BACKGROUND

As the population ages, health care can become more complicated because of multimorbidity and polypharmacy.^1^ Older patients’ health problems may also be affected by their culture and habits.^2^ Comprehensive care that considers how patients relate to their medical conditions is necessary to improve the state of health care today.^3^ For comprehensive care, various medical professionals need to share patients’ information. Organ‐specific specialists may have difficulty in dealing with patient issues outside of their specialty, so generalists with skills in managing complicated cases are required.^4^ General physicians have a broad range of medical knowledge and skills that can be applied to various medical problems. Additionally, generalists are competent in interprofessional collaborations, and they may become facilitators in allied professional partnerships.^5^ Increasing the number of general physicians may alleviate key healthcare concerns of aging societies.

Establishing a general physician educational system that appeals to medical students and residents is essential. Despite this need, in recent years and especially in Japan, most medical students have become organ‐specific specialists and not general physicians.^6^, ^7^ To increase the number of general physicians, well‐established educational programs are essential.^8^ However, medical students may not have many opportunities to be exposed to general medicine because there is a lack of educators who are general physicians in well‐established medical programs.

For students in medical universities, hands‐on training in community hospitals and clinics can be an effective pedagogical tool to learn more about general medicine.^7^, ^9^ Through training in these settings, medical students can learn the various competencies required to be a general physician. Furthermore, in these clinical settings, the students can expand on their knowledge of general medicine obtained in medical universities.^7^ Improved training in community medical institutes could be the key to increasing the number of general physicians. It has been observed that medical students are more likely to become general physicians after training in communities during their undergraduate and postgraduate education.^10^, ^11^ This trend may be stronger when educational settings are in rural communities because students can experience the essence of general medicine such as patient‐centered medicine, interprofessional collaboration, and community‐oriented primary care.^12^, ^13^ Through these experiences, medical students may get a true sense of the importance of general physicians, which may motivate them to become general physicians.

Although education in community hospitals and clinics is prevalent, medical students in Japan may not be appropriately educated about general medicine as only a small percentage seek residencies in this area.^7^ There is a variety of evidence about the effects of primary care/family medicine/general medicine education on students’ perceptions of general physicians or their professional choices in universities outside of Japan.^14^, ^15^ However, few studies have clarified how general medicine education in community hospitals or clinics affects students’ perceptions of general physicians or their professional choices in Japan. This clarification could lead to a better understanding of the current situation and guide further interventions to promote general medicine education. The purpose of this research was to determine medical students’ perceptions about general medicine in Japan, and the advantages and disadvantages of general medicine training in a community hospital based on their participation in a community‐based medical education program (CBME). This information is necessary to improve general medicine education in Japan and in other countries that face similar shortages of primary care physicians.

## 2 MATERIALS AND METHODS

### 2.1 Method and research design

To inquire about the effect of education and medical students’ perceptions of general medicine in Japan, we chose to employ a mixed‐method design. Quantitative data were obtained from survey questionnaire responses, and qualitative data were derived from semistructured interviews.

### 2.2 Setting

#### 2.2.1 The condition of general medicine education in Shimane, Japan

In Shimane, Japan, general medicine education is conducted in Shimane Medical University, community hospitals, and clinics. In the medical students’ fifth year, mandatory exposure to clinical settings of general medicine comprises two weeks in community hospitals or clinics. In the sixth year, the duration increases to one‐ to two month training programs. At the university, general physicians only work in emergency rooms, and they do not regularly visit clinical settings. General physicians at community hospitals and clinics cover various medical problems, including those related to pediatric and geriatric patients.

#### 2.2.2 Unnan City and hospital

Unnan is one of most rural cities in Japan, located in the southeast of Shimane Prefecture. In 2017, the total population of Unnan was 38 882 (18 720 males and 20 162 females), its aging rate is 37.82%, and this is expected to reach 50% by 2025. Each family lives separately. In Unnan, there are 16 clinics, 12 home care stations, three visiting nurse stations, and only a single public hospital (Unnan City Hospital). At the time of the study, Unnan City Hospital had 281 beds comprising 160 acute care beds, 43 comprehensive care beds, 30 rehabilitation beds, and 48 chronic care beds. There were 14 medical specialties, and the nurse‐to‐patient ratio was 1:10 in acute care, 1:13 in comprehensive care, 1:15 in rehabilitation, and 1:25 in chronic care. There were physicians in the hospital who were specialized in family medicine/primary care. These physicians treated patients with multiple diseases in inpatient and outpatient situations. All of them engaged in the education of medical students and residents.

### 2.3 Participants

As part of the mandatory curriculum at their Japanese medical university, fifth year and sixth year undergraduate medical students undertake a two week rotation with Unnan City Hospital to learn about rural medicine. Before this training experience, students finished studying the fundamentals of clinical medicine and passed a computer‐based test. Additionally, they completed an objective, structured mandatory clinical examination to participate in patient care. Between April 2018 and March 2019, 15 medical students participated in the CBME curriculum with Unnan City Hospital. Of the 15 participants, eight were fifth year students, and seven were sixth year students. The average age was 24.8 years old (SD = 3.2), and six were male.

#### 2.3.1 Ethical considerations

Before beginning the course, we explained this research project to the students and obtained informed written consent. The participants were informed that they could withdraw their participation at any time, for any reason, without repercussions. They were informed that participation was voluntary, their responses confidential, and the interview information was accessible only to the study authors. The research was approved by the Unnan City Hospital Clinical Ethics Committee.

### 2.4 Community‐based medical education (CBME) course in Unnan

The participants took part in a two week CBME course in Unnan, focusing on general medicine or family medicine through a variety of learning environments. The main learning situations were chosen with respect to competencies required in Japanese general medicine: person‐centered care, comprehensive/integrative approach, interprofessional work, community orientation, professionalism, and system‐based practices. Each competency was incorporated into the two week schedule (Table 1). The students worked with family physicians in hospitals (including emergency rooms) and clinics to learn about common diseases and illnesses. They collaborated with care managers and home care workers to learn about interprofessional work. In the community care settings, the students participated in community activities such as discussions about citizens’ health to learn about the reality of rural citizens’ lives from the perspective of person‐centered care and community orientation.^16^ At the end of each day, the students were asked to reflect on the day's activities to promote self‐determination professionalism in learning. As part of this reflective practice, students were given time to present what they had learned in the different medical and community settings using the flipped classroom instructional strategy.

**Table 1:** A sample of the learning content

	Monday	Tuesday	Wednesday	Thursday	Friday
Week 1					
Morning	Outpatient (community hospital)	Outpatient (community hospital)	Community care	Clinic	Outpatient (community hospital)
Afternoon	Inpatient (community hospital)	Emergency room	Home visit	Clinic	Inpatient (community hospital)
Week 2					
Morning	Home care worker	Outpatient (community hospital)	Care manager	Clinic	Outpatient (community hospital)
Afternoon	Home care worker	Inpatient (community hospital)	Care manager	Clinic	Inpatient (community hospital)

### 2.5 Instruments and interviews for collecting data

Our achievement questionnaire (Table 2) was determined to be valid and reliable in the Japanese context based on previous CBME assessment reports.^17^, ^18^ The participants rated ten items using a five‐point Likert scale (1 = fully disagree, 5 = fully agree) before and after completing the two week CBME course in Unnan. A second survey questionnaire was developed to assess the students’ perceptions of curriculum quality. The participants completed the curriculum quality survey (Table 3) after completing the two week training, again using a five‐point Likert scale (1 = fully disagree and 5 = fully agree). Ratings of >3.5 met the curriculum objectives, as suggested by the previous research.^18^

**Table 2:** Student responses on achievement questionnaire before and after CBME course

	Content	Before		After		*P*‐value
		Mean	95% CI	Mean	95% CI	
1	I can explain the function of general medicine.	2.54	2.22‐2.85	3.4	3.12‐3.68	<.001
2	I can explain the difference in the functions of tertiary hospitals and community hospitals.	2.46	2.06‐2.86	3.13	2.85‐3.42	.006
3	I can explain the importance of solving each patient's problems based on the biopsychosocial model.	2.77	2.41‐3.13	3.53	3.25‐3.82	<.001
4	I can explain the relationship between patients’ health conditions and their backgrounds.	2.61	2.22‐3.01	3.07	2.81‐3.32	.041
5	I can take down history and conduct physical examinations properly.	2.23	1.79‐2.67	3	2.79‐3.21	<.001
6	I can explain the importance of preventable medicine to citizens.	2.38	1.86‐2.91	3.2	2.97‐3.43	.003
7	I can explain the jobs of care managers.	2.23	1.87‐2.59	3.47	3.18‐3.75	<.001
8	I can explain the jobs of care workers.	2.23	1.87‐2.59	3.33	2.99‐3.68	<.001
9	I can explain the functions of home care.	2.54	2.22‐2.85	3.07	2.92‐3.21	.002
10	I can explain the importance of dialogue with citizens.	3	2.57‐3.43	3.47	3.11‐3.82	.078

Abbreviations: CBME, community‐based medical education; CI, confidence interval.

**Table 3:** Curriculum content survey responses

	Contents	Mean	SD
1	The learning content was useful for my career.	4	0.67
2	The learning content was relevant to what I learned at university.	3.11	0.94
3	The curriculum purposes were clear.	4.68	0.48
4	The curriculum drove my self‐directed learning.	4.89	0.32
5	The subjects given were relevant to the curriculum purposes.	4.63	0.49
6	Reflection was useful for my learning.	4.16	0.96
7	The learning about general medicine was useful for my future.	4.89	0.32
8	The dialogue with citizens was meaningful.	4.16	0.76
9	The curriculum content was appropriate.	3.63	0.59
10	The quality of the educational institution was desirable.	4.68	0.48
11	I could share my learning with my teachers.	4.44	0.59
12	I am motivated to be a general physician.	4.47	0.51

Abbreviation: SD, standard deviation.

We conducted one‐on‐one 30 minute semistructured interviews with participants soon after they completed the postcourse questionnaires. The interviews followed a topic guide that included: “the advantages of this curriculum,” “the disadvantages of this curriculum,” “perceptions of general physicians and how these changed through the learning process,” and “restrictions on learning about general medicine in throughout their curriculum, including in their universities.”

### 2.6 Analysis

We used paired t tests to assess the changes between pre‐ and postlearning achievements in the questionnaire responses. To analyze the content of the one‐on‐one interviews, we used a thematic analysis.^7^ The analysis comprised familiarization with the data, generation of the initial codes, searching for themes, reviewing the themes, defining and naming the categories, and producing a report that includes the selection of exemplification data and quotations.^19^ All of the interview content was transcribed verbatim, coded independently by R.O. and Y.R., and then, the open coding was checked for agreement. The researchers then discussed the open codes and emerging concepts, and where disagreements occurred, they recoded or redefined the concepts and categories. The data collection and analysis were conducted iteratively, and the data collection continued until no new concepts emerged. To minimize personal bias, the transition from the codes to the preliminary themes and then to the final themes included frequent discussions between the two authors.

## 3 RESULTS

Table 1 illustrates the ratings for the achievement questionnaire administered before and after the CBME course and the statistical comparison of the change in the mean group scores (*t* test). On the achievement questionnaire, the ratings significantly improved (*P* < .05) for nine out of 10 questions (item 10 *P* = .078). Table 3 illustrates the results for the curriculum contents survey, administered only after the 2 week CBME course. The ratings were more than 3.5 for 11 of the 12 items (exception is item 2: *The learning content is relevant to what I learned in the university*).

The thematic analysis revealed the participants’ perceptions regarding curriculum advantages, disadvantages, and general physicians. The two themes (driving forces for general medicine and roadblocks from multiple perspectives) and their 11 related concepts are illustrated with the students’ quotes in Table 4.

**Table 4:** Themes and concepts: Students’ perceptions about general medicine post‐CBME course

Theme	Concept	Quote
Driving forces for general medicine	Acquisition of various perspectives	“Home care professionals had different perspectives from medical doctors. Their jobs were deeply involved with patients’ lives, and their activities matched the patients’ needs. I knew the knowledge, but in reality, it was impressive, and physicians should have that perspective.” (Student 3)
	Realization of the physician's role	“Medical matters may be difficult for other medical professionals. If we can mitigate those difficulties, the collaboration with them becomes more effective, and this may be vital for patients’ care.” (Student 2)
	Importance of community care	“Usually, I would see citizens at my university hospital, but their mannerisms within their communities were completely different from at the hospital. They were relaxed and natural, and they had their own lifestyles. Their lifestyles may be essential to their health. That part requires education.” (Student 1)
	Respect for individuals’ lifestyles	“Some citizens have bad health habits, such as eating high‐calorie food, drinking alcohol, and not exercising. They should be reviewed independently but may be affected by their culture and context. These factors cannot be changed effectively, so we should respect their lifestyles.” (Student 8)
	Approach to multimorbidity	“I was impressed that general physicians were able to deal with many different kinds of medical conditions regardless of the problem that presented itself. Their medicine is cool and meaningful in aging societies. This is one of the appealing points for motivating medical students and residents to become general physicians.” (Student 10)
	Variety of settings	“General physicians are able to work in various clinical settings and, in reality, I felt that their work was both needed and respected by citizens. As I had never expected such work, I was impressed that general medicine is suitable for the needs of rural medicine.” (Student 7)
	Collaboration with various professionals	“By learning more about the content of other professions, I understood the importance of general medicine. In the care of patients with complex needs, physicians have to know various aspects of their patients’ care and both facilitate and lead their care team. General physicians can do this effectively because they know various issues regarding medicine and have collaborative skills.” (Student 9)
Roadblocks from multiple perspectives	Lack of exposure	“Here, I realized the importance of general medicine and was motivated to be a general physician. Having said that, in the existing conditions, medical students may lack the exposure to general medicine because of insufficient education at our university. If they do not go to hospitals where general physicians work, they may not experience the effectiveness and worthiness of general medicine.” (Student 14)
	Ignorance from other specialists	“Other medical specialists do not understand the function of general physicians. At our university, we had a different way of learning about general medicine compared to here. Not just me, but also other students may not know the proper functions of general medicine. This may not lead to an increase in the number of general physicians.” (Student 13)
	Imprecise domain	“General medicine is interesting for me, but I cannot clearly tell other medical students what general physicians are able to do. If there were a clear blueprint for general physicians, they would have a better reputation with medical professionals and students.” (Student 1)
	Bias against rural medicine	“We learned about general medicine only in the context of rural medicine. However, we do not know anything about rural medicine itself, so many students may be confused and not motivated to learn general medicine. If we can be informed about general medicine and rural medicine properly, we may be motivated.” (Student 4)

Abbreviation: CBME, community‐based medical education.

### 3.1 Driving forces for general medicine

#### 3.1.1 Acquiring various perspectives

Through interactions with a variety of professionals and citizens, such as home care workers, care managers, and home care nurses with whom the participants had not taken classes at their university, the participants were able to learn how other medical professionals think about patients. The participants were impressed that other professionals focused not only on medical issues but also on patients’ everyday lives. The participants learned about comprehensive medical care in hospitals and their homes, motivating them to learn patient‐centered care.

#### 3.1.2 Realization of the physicians’ role

The participants of this CBME learned about their future roles for effective interprofessional collaborations. Through communications with multiple professionals, the students realized that all medical professionals have expertise, but this expertise is different from the medical knowledge of general physicians.

#### 3.1.3 Importance of community care

The students were exposed to various aspects of the community residents’ lives through this curriculum. In medical institutions, patients generally adhered to physicians’ suggestions. However, in their communities, the individuals insisted on their own ideas and acted independently to improve their lives and health. As the participants had only previously communicated with patients in their medical institution, they perceived individuals in their medical institutions differently than those in their communities. Outside of medical institutions, the participants learned how different lifestyles affect health, and these can be modified with community care.

#### 3.1.4 Respect for different lifestyles

The community members had different lifestyles based on cultural and individual differences. Through interactions with the community residents, the participants were able to understand the lives of patients in actuality as against what they had learned at university. Through various interactions, the students learned about community‐oriented primary care and patient‐centered care.

#### 3.1.5 Approach to multimorbidity

For the participants, an exciting aspect of general medicine was the need to consider various medical conditions that occurred simultaneously in one patient. General physicians were able to analyze their patients’ medical conditions and make several plans for each medical problem. This was novel for the participants as they experienced organ‐specific physician care in their university. General physicians’ approaches to patients with multimorbidity impressed medical students, and it should be emphasized as an area of general physician competency.

#### 3.1.6 Variety of settings

The participants experienced general medicine in various settings, such as outpatient, inpatient, home care, and community care. In each setting, general physicians functioned to sustain and improve patients’ health. The participants found general physicians’ competence in a variety of settings very appealing, and this diversity could motivate them to become general physicians.

#### 3.1.7 Collaboration with various professionals

The study participants collaborated with a variety of medical professionals. They understood the significance of general physicians’ skill in collaborating with other medical professionals. Professional collaborations drove the quality of care for patients with multimorbidity issues or requiring home care, and general physicians directed these collaborations. The participants learned about the efficacy of collaboration among medical professionals as a core competency of general physicians.

### 3.2 Roadblocks from multiple perspectives

#### 3.2.1 Lack of exposure

This CBME course was the students’ first real exposure to the general medicine field. The low number of general physicians is attributed to a lack of student understanding regarding their role. Increasing general physicians’ role in medical education in both community hospitals and universities seems key to increasing the recognition of general medicine among medical students.

#### 3.2.2 Ignorance from other specialists

In the university setting, students usually learn about general medicine from other medical specialists. In interviews, the participants suggested that medical professionals at university and urban general hospitals misunderstood or were ignorant about the functions and abilities of general physicians. This misinformation potentially deters students from learning about general medicine or choosing it for their future career.

#### 3.2.3 Imprecise domain

During the interviews, the participants suggested that general medicine could be practiced by many physicians, especially in rural areas. General medicine has significant potential, but its opaqueness may be a roadblock to increasing the number of general physicians.

#### 3.2.4 Bias against rural medicine

The participants had various impressions of rural medicine. During their university program, they were taught about rural medicine and encouraged to work at rural medical institutions. Their understanding and biases against rural medicine caused them to avoid general medicine. Information about educational settings from their university programs affected medical students’ perceptions, making them reluctant to learn about general medicine.

## 4 DISCUSSION

In this study, 15 medical students learned about the functions of general physicians and allied professionals, and the importance of interprofessional and community collaborations during a two week CBME program in a small city in rural Japan. The content of the educational curriculum was assessed positively by the medical students, except for the continuity of learning between medical universities and community hospitals. Additionally, the participants described the issues regarding general medicine, especially about the difficulties in learning general medicine at their universities. The participants found various aspects of general medicine appealing, but the ignorance of the functions of generalists among other medical professionals as well as the opaqueness of the general physician's role deterred students.

Differences in the achievement questionnaire before and after the CBME experience demonstrated that the participants learned about rural medicine, general medicine, and interprofessional collaborations and were more motivated to learn about general medicine. There were significant positive changes in nine of ten areas. Item 10 (“the importance of dialogue with community members”) did not improve statistically. However, item 10 had the highest pretest and posttest rating; thus, it might be that the small sample size was statistically insufficient to detect significant change. Although the participants were interested in talking with community members, they had only one day to do so.^20^, ^21^ As the students are exposed to patients in medical institutions and communities, they might be able to realize the importance of community care and respect for their lives.

The participants’ ratings of the curriculum content indicated that most parts met the CBME objectives (Table 2). The one item rated less than adequate was “the learning content is relevant to what I learned in the university” (mean rating = 3.11). This rating indicated relatively poor learning continuity between the community and university hospitals. Unnan City Hospital's curriculum was also based on the competency of general medicine. Our curriculum is new, and revisions are necessary to improve the medical students’ learning, such as an introduction to the general medicine related to what they had learned at their universities. Furthermore, based on the qualitative analysis of the interview data, there may be real discrepancies in general medicine education between community hospitals and medical universities. Generally speaking, primary/general medicine is difficult to teach only in universities and should also be taught in community hospitals and clinics where continuity of care, patient‐centered care, and community‐oriented primary care can be emphasized.^22^, ^23^ For effective learning, medical universities and community hospitals need to collaborate and share the content of their educational programs.^24^ In Japan, education about general medicine has started only recently, and collaborations between educational programs may be negligent.^7^, ^25^, ^26^ The next step in Japanese general medicine education is to solidify connections between community hospitals and medical universities regarding CBME.

The qualitative data analysis revealed various restrictions on the education of general medicine and primary care. One issue was the medical students’ lack of exposure to general medicine. In Japanese undergraduate medical education, rural medicine, primary care, and general medicine education are mandatory.^27^ However, medical students may not be able to learn enough about them in the present system, even in other countries.^28^, ^29^ Ignorance from other specialists and the imprecise domain of general medicine may also inhibit effective teaching about general medicine in Japanese universities. The exchange of medical teachers between universities and community hospitals could enhance understanding of the diverse conditions in which physicians practice.^30^

Approaches to multimorbidity and interprofessional collaboration in various settings appeal to medical students. General medicine is one of the most versatile medical fields, and practitioners are required in all settings.^8^ As medical students are flexible and adept at learning various topics, multitasking might stimulate their interests. 23 Additionally, in this curriculum, the medical students learned about other professionals in medical institutions and communities. From this, they might be able to broaden their perspectives about their patients’ real lives.^20^

## 5 CONCLUSION

There are several study limitations. We provided this new curriculum to a relatively small group of medical students (n = 15). However, despite this small sample size, most pre‐/posttraining comparisons improved statistically, indicating the effectiveness of our curriculum. We added a qualitative analysis to inquire about the medical students’ perceptions about general medicine. The transferability of the students’ perceptions regarding general medicine is another study limitation. However, to sustain quality, we interviewed all study participants and coded to saturation.

This study demonstrates that CBME can improve students’ learning and interest in general medicine. However, there may be gaps between education in medical universities and community hospitals regarding general medicine. General medicine competencies, such as approaches to multimorbidity and interprofessional collaborations in various settings, should be taught, and future studies should determine the curriculum outcomes in general medicine, by focusing on these competencies. Additionally, data on the current understanding of general medicine by other medical specialists are required, and there should be continuity and cooperation between medical universities and community hospitals. These combined efforts are indispensable in improving the quality of medical education.

## CONFLICTS OF INTEREST

The authors have stated explicitly that there are no conflicts of interest in connection with this article.
